# Visible minority status and occupation were associated with increased COVID-19 infection in Greater Vancouver British Columbia between June and November 2020: an ecological study

**DOI:** 10.3389/fpubh.2024.1336038

**Published:** 2024-02-28

**Authors:** Binay Adhikari, Younathan Abdia, Notice Ringa, Felicity Clemens, Sunny Mak, Caren Rose, Naveed Z. Janjua, Michael Otterstatter, Michael A. Irvine

**Affiliations:** ^1^BC Centre for Disease Control, Vancouver, BC, Canada; ^2^School of Population and Public Health, University of British Columbia, Vancouver, BC, Canada; ^3^Faculty of Health Sciences, Simon Fraser University, Burnaby, BC, Canada

**Keywords:** Bayesian analysis, health disparities, social determinants of health, COVID-19 pandemic, geospatial analysis and modeling, visible minority groups, epidemiology

## Abstract

**Background:**

The COVID-19 pandemic has highlighted health disparities, especially among specific population groups. This study examines the spatial relationship between the proportion of visible minorities (VM), occupation types and COVID-19 infection in the Greater Vancouver region of British Columbia, Canada.

**Methods:**

Provincial COVID-19 case data between June 24, 2020, and November 7, 2020, were aggregated by census dissemination area and linked with sociodemographic data from the Canadian 2016 census. Bayesian spatial Poisson regression models were used to examine the association between proportion of visible minorities, occupation types and COVID-19 infection. Models were adjusted for COVID-19 testing rates and other sociodemographic factors. Relative risk (RR) and 95% Credible Intervals (95% CrI) were calculated.

**Results:**

We found an inverse relationship between the proportion of the Chinese population and risk of COVID-19 infection (RR = 0.98 95% CrI = 0.96, 0.99), whereas an increased risk was observed for the proportions of the South Asian group (RR = 1.10, 95% CrI = 1.08, 1.12), and Other Visible Minority group (RR = 1.06, 95% CrI = 1.04, 1.08). Similarly, a higher proportion of frontline workers (RR = 1.05, 95% CrI = 1.04, 1.07) was associated with higher infection risk compared to non-frontline.

**Conclusion:**

Despite adjustments for testing, housing, occupation, and other social economic status variables, there is still a substantial association between the proportion of visible minorities, occupation types, and the risk of acquiring COVID-19 infection in British Columbia. This ecological analysis highlights the existing disparities in the burden of diseases among different visible minority populations and occupation types.

## 1 Introduction

The effect of the COVID-19 pandemic disproportionately impacted different demographic sub-groups. This disproportionate effect is largely attributed to the existing inequalities across different levels of social and economic status (SES) (1, 2). For instance, there are well-documented associations between COVID-19 cases and ethnicity, socio-economic background, and occupation. Pre-existing health conditions, such as obesity, diabetes, and cardiovascular diseases, also tend to be higher among vulnerable populations, thereby creating a disproportionate distribution of risk of severe outcomes after COVID-19 infection (3). To address the impact of the pandemic, it is therefore important to understand the role of different socio-economic factors on COVID-19 infections.

Racially biased policies have had detrimental effects on access to healthcare and opportunities for employment, leading to differences in COVID-19 incidence (2). Different studies from the United States examining the disease pattern across different ethnicities have shown higher rates of infection and mortality associated with Hispanic and black minorities (4–6). Similar findings among South Asian populations were found in various studies in the UK (3, 7, 8), suggesting a higher risk of COVID-19 infection among visible minority ethnic groups.

In addition to racial and ethnic factors, certain occupation types are associated with a higher risk of COVID-19 infection (9, 10). It became evident from the early study of the pandemic that persons employed in healthcare-related occupations had a higher risk of infection (11). Other high infection risk occupations include those categorized as frontline services, such as those related to the food, safety, transportation, and manufacturing industries. During the pandemic there was a significant shift to remote work to minimize physical contact; however, certain frontline services continued to operate as usual. As many frontline sector roles were not amenable to remote work, persons in these roles were at increased risk of exposure (12–14).

Due to the spatial correlation of ethnicity and occupation type within geographic areas, specialized statistical methods need to be applied to discern the relationship between these factors and disease incidence. Regression methods, including multiple piecewise linear regression models and nonlinear regression models, including generalized additive models and generalized additive mixed models, have been widely used to study the dose relationship between explanatory variables and outcomes relevant to COVID-19, such as infection, severity, and mortality (15). However, conventional regression methods ignore the spatial dependence between the geographical units under study, which is essential to understanding relationships for infectious disease. As a result, various studies have been developed to analyze the spatial determinants of the spread of COVID-19 across different geographies (16–19).

Our contribution in this study is to estimate the association between visible minority groups, occupation, and COVID-19 cases, accounting for the spatial dependency between these factors within a large urban center in North America. We hypothesize that higher proportion of visible minority population and population in frontline occupations would be associated with an increased risk of COVID-19 infection, controlling for area-level sociodemographic indicators. We test this hypothesis by examining the relationship between proportion of visible minorities, occupation types, and COVID-19 infection using an ecological approach. This allows us to account for the neighborhood level risk factors associated with COVID-19 infection, albeit not at an individual level. We further explore which SES factors are associated with an increased risk of SARS-CoV-2 infection at a neighborhood level. We test these hypotheses using a series of Bayesian Poisson spatial regression models with covariates adapted to each hypothesis explored. We also control for COVID-19 testing rates to account for possible differences in test-seeking behavior and barriers to testing, which can result in systematic undercounting (20–22).

## 2 Methods

### 2.1 Study context

This study was conducted in the Greater Vancouver region in the lower mainland area of British Columbia, Canada, the 3rd largest metropolitan region of Canada (2021 census population of 2.9 million). This region includes the Metro Vancouver Regional District and parts of the Fraser Valley Regional District, which has wide variation in its socio-demographic composition, including multiple ethnicities and occupations.

We limited our study timeline from June 24, 2020, to November 7, 2020, to include a period of broader societal ‘lockdown’, re-opening following initial restrictions, and the start of the school year in September. This provides a setting well suited to studying community COVID-19 transmission using neighborhood-level determinants of health. We excluded cases in high-exposure risk settings (i.e., cases among residents of long-term care and assisted living facilities and correctional facilities).

### 2.2 Study design and data sources

We used an ecological design to study the relationship between proportion of visible minorities, occupation types, and COVID-19 infection risks using Census Dissemination Area (DA) level data.

The COVID-19 infection data were aggregated at the Census Dissemination Area (DA) level and linked with the 2016 census data. The visible minorities in our study population are based on the definition in the Employment Equity Act of Canada as persons other than Aboriginal peoples, who are non-Caucasians in race or non-White in color. Statistics Canada uses this definition while collecting census data on visible minority status. This visible minority group comprises of South Asian, Chinese, Black, Filipino, Arab, Latin American, Southeast Asian, West Asian, Korean, and Japanese (23).

### 2.3 Outcome variable

The number of COVID-19 infections was measured using an integrated COVID-19 laboratory dataset, containing details about COVID-19 PCR-based laboratory tests conducted by private and public laboratories across BC. Positive laboratory results were aggregated at the census Dissemination Area (DA).

These counts of infections by DA were standardized to give the Standardized Incidence Ratio for descriptive analysis, as noted below.

### 2.4 Exposure variables

The exposure variables included proportion of visible minority populations and proportion of different occupation types based on the 2016 census. Visible minorities included South Asian, Chinese, Southeast Asian, Filipino, Korean, Japanese, Black, Arab, West Asian, Latin American, and others. Occupation types were based on the National Occupational Classification (NOC) (24) and comprised trade, transport, and equipment; sales and services; natural and applied science; manufacturing and utilities; management; healthcare; education, law and government; business, finance and admin; natural resource, agriculture, and production; and art, culture, and sports. Occupation was categorized as frontline, non-frontline, and unemployed. Frontline work included occupations not amenable to remote work settings, i.e., sales & services, trade/transport/equipment operator, natural resources/agriculture, manufacturing/utilities, and healthcare. Non-frontline occupations included occupations related to management, business/finance/administration, natural/applied sciences, education/law/government, and art/culture/sports, which could be done remotely, which, in turn, lessens the risk of COVID exposure.

### 2.5 Confounding factors

The socio-demographic variables adjusted for in these analyses included: average family size and the proportion of suitable housing (based on the National Occupancy Standard), the proportion of recent immigrants (arrived in Canada in 2011 or afterwards but before the 2016 census), the proportion of population using public transport to commute to work, the unemployment rate (reported as a proportion), and the proportion of males. We used prior literature as a reference for determining these confounding factors (12, 25–27).

For more details about socio demographic variables, see Supplementary Table S1.

In addition, we incorporated the total SARS-CoV-2 PCR-based tests *per capita* in each DA.

### 2.6 Statistical methods

#### 2.6.1 Descriptive analysis

We mapped the distribution of the population risk of COVID-19 per 100,000 by DA. We then summarized the composition of the population under study with respect to socio-demographic variables across all DAs by pooling the proportions and reporting the mean, standard deviation, and quartiles for these pooled metrics. Average family size was pooled across all DAs and reported using mean, median, standard deviation, minimum, maximum, and quartiles.

We summarized the geographic distribution of cases within each DA using the Standardized Incidence Ratio (SIR). The observed number of cases within each DA was described above. Expected counts of cases were calculated using 20 strata based on age and sex distributions for the study region and using indirect standardization. We then calculated the SIR for each DA as the ratio of observed number of cases to expected number of cases.

#### 2.6.2 Inferential analysis

The outcome for this analysis was counts of positive SARS-CoV-2 PCR-based tests by DA across the study period, which were assumed to be Poisson-distributed conditional on DA covariates, testing rate, and inferred geospatial covariance. The approach used was that of Bayesian geospatial Poisson modeling for counts. We extended the Bayesian Besag-York-Mollié (BYM) (28) geospatial model on the risk ratio of a reported COVID-19 case for each DA (29, 30). A Bayesian statistical approach was used to incorporate unstructured and spatially structured heterogeneity into the model. Model estimates were provided as medians of the posterior sample, with uncertainty quantified using 95% credible intervals.

We modeled the log relative risk as a sum of key exposure variables, socio demographic covariates and spatially structured and unstructured random variates. Spatially structured errors were modeled as Intrinsic Gaussian conditional autoregressive (ICAR) errors (31). Unstructured errors were specified as independent Gaussian random variables with gamma-distributed variance. We estimate the exceedance probability as the posterior probability that the relative risk within a DA is greater than the 75th percentile of all DA regions.

All Bayesian priors were chosen to be weakly informative, setting the scale of the model coefficients to epidemiologically plausible ranges of the relative risk (32).

#### 2.6.3 Adaptations to the model to account for variable testing rates

The Bayesian model incorporated a logit probability of detection using a fixed intercept and the log-test rate multiplied by a test rate parameter. This estimated probability of detection was multiplied by the total expected rate in order to adjust for variable testing rates across the DAs. As a sensitivity analysis, linear test rates were used instead of log test rates and found to provide similar marginal posterior estimates for the SES coefficients. Additionally, a separate model (the unadjusted testing model) was run where the probability of testing was fixed for all regions.

#### 2.6.4 Supplementary analyses using sub-components of visible minority and occupation types

Separate models including different visible minorities and frontline occupations were run to examine the differences in infection risk among different sub-groups.

All models were performed using R version 4.1.0 and the Nimble R package, which implements an adaptive Metropolis-Hastings Markov Chain Monte Carlo scheme across 1,000 iterations with 1,000 burn-in iterations across four chains and no thinning. Model convergence was checked using the Gelman-Rubin statistic and by visible inspection of the chains for a random selection of variables. Further model output is provided in the accompanying Shiny dashboard application.^1^

This work was unfunded and conducted under a public health surveillance mandate.

## 3 Results

The final analyses included 3,860 DAs after removal of 5 DAs due to missing data as they did not meet data suppression threshold for suppression used by Statistics Canada. The threshold for suppression is 40 persons for shortform questions (e.g., age, sex, marital status, etc.), and 250 persons for longform questions (e.g., ethnicity, income, education, etc.)

**Figure 1:**
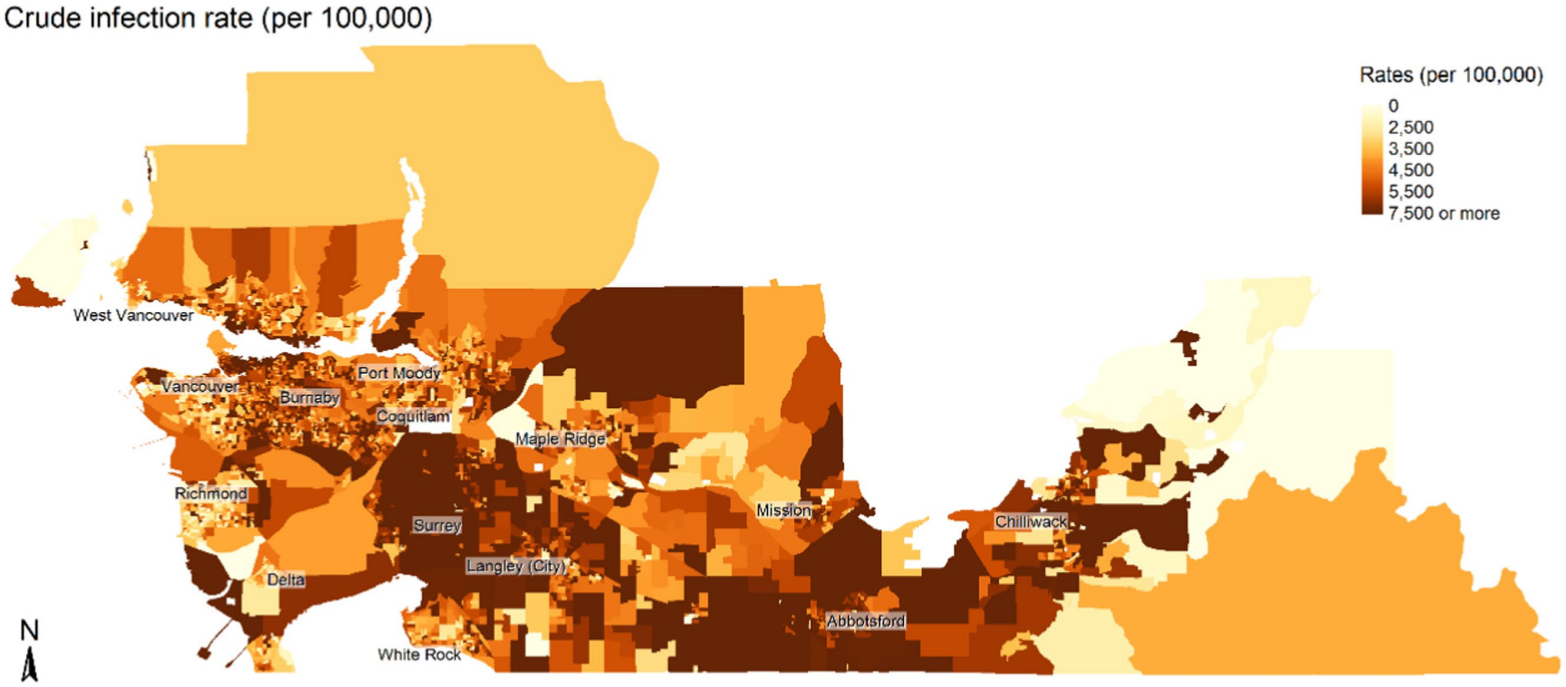
Spatial distribution of the crude rate of COVID-19 infection per 100,000 population over the study period (June and November 2020). Map is not to scale.

**Figure 2:**
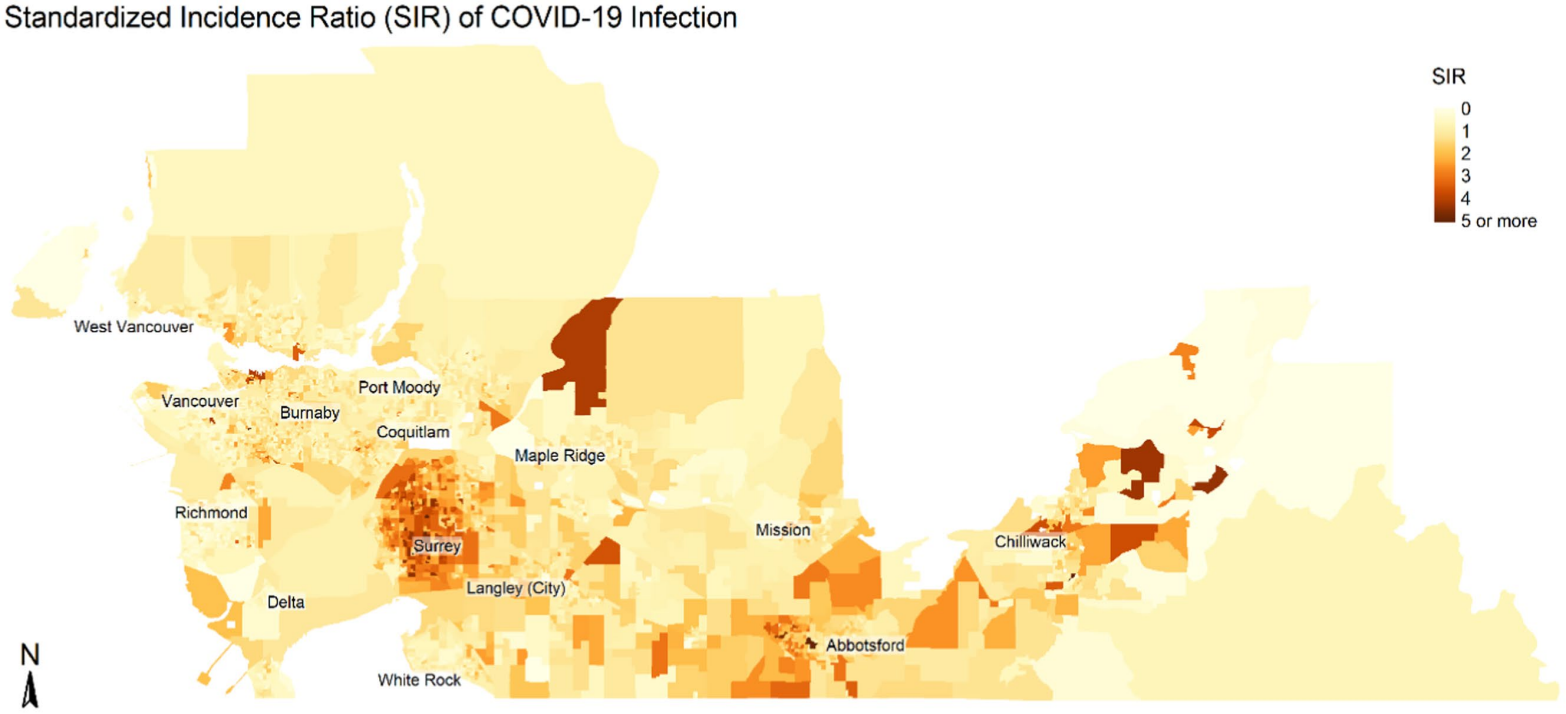
Spatial distribution of the Standardized Incidence Ratio (SIR) of COVID-19 infection. SIR shows the ratio between expected counts and observed counts of COVID infections based on population by age and sex in each census dissemination area. Map is not to scale.

Tables 1, 2 summarize the socio-demographic characteristics of the DAs used in the study. Figures 1, 2 show the spatial distribution of COVID-19 infection across the study region. As seen in Figures 1, 2, there was a higher concentration of COVID-19 infection in southeastern areas of the study region. DAs in Surrey municipality and parts of Abbotsford and Chilliwack municipalities show higher incidence rates. Some DAs in Vancouver municipality also showed a higher infection rate. Similarly, there were pockets of areas spread across the study region with higher incidence rates of COVID-19 infection.

**Table 1:** Summary of key exposures, covariates, and outcome variables at the dissemination area level from Statistics Canada.

Variable	Metric used*	N^‡^	Median	Mean	Sd	Min	Pctl. 25^‡^	Pctl. 75^‡^	Max
Key exposures									
Visible minority									
South Asian	Proportion	3,860	0.04	0.11	0.18	0.00	0.02	0.11	1.00
Chinese	Proportion	3,860	0.09	0.18	0.20	0.00	0.03	0.28	1.00
Other visible minorities	Proportion	3,860	0.12	0.15	0.11	0.00	0.07	0.21	0.69
Frontline workers^†^	Proportion	3,860	0.48	0.49	0.14	0.00	0.39	0.58	1.00
Covariates									
Average family size	Average no of people/household	3,860	2.80	2.70	0.62	1.00	2.30	3.10	5.20
Recent immigrants	Proportion	3,860	0.04	0.05	0.05	0.00	0.02	0.07	0.41
Suitable housing	Proportion	3,860	0.95	0.93	0.07	0.00	0.90	0.98	1.10
Public transport users	Proportion	3,860	0.16	0.18	0.13	0.00	0.08	0.27	0.81
Male proportion	Proportion	3,860	0.49	0.49	0.03	0.27	0.47	0.51	0.81
Unemployment rate	Proportion	3,860	0.06	0.06	0.04	0.00	0.04	0.08	0.33
Test rate	Proportion	3,860	0.13	0.20	1.10	0.00	0.10	0.18	63.00
Outcome									
Case rate	Proportion	3,860	0.04	0.05	0.03	0.00	0.02	0.05	0.37

^*^Metrics were pooled from all DAs and summarized. ^‡^N = Number of Dissemination Area with non-missing information; Pctl. 25 = 25th percentile, Pctl. 75 = 75th percentile. ˟Other visible minorities include Black, Filipino, Latin American, Arab, Southeast Asian, West Asian, Korean, Japanese, Multiple Visible Minorities (it includes those who gave more than one visible minority responses), and Visible minority, n.i.e (it includes person who provided ethnicity with write-in response). See Supplementary Table S1 for further information. ^†^Frontline worker includes occupations related to sales & services, trade & transport, natural resources & agriculture, manufacturing & utilities, and healthcare workers based on the National Occupation Classification (NOC). See Supplementary Table S1 for further information.

**Table 2:** Summary of sub-categories of visible minorities and occupation types from Statistics Canada used as predictors for the supplementary analyses.

Variable	Metric used*	N^‡^	Median	Mean	Sd	Min	Pctl. 25^‡^	Pctl. 75^‡^	Max
Visible minority subcategories˟									
Black	Proportion	3,860	0.00	0.01	0.02	0.00	0.00	0.02	0.30
Filipino	Proportion	3,860	0.02	0.04	0.06	0.00	0.00	0.06	0.45
Latin American	Proportion	3,860	0.00	0.01	0.02	0.00	0.00	0.02	0.18
Arab	Proportion	3,860	0.00	0.01	0.02	0.00	0.00	0.00	0.32
Southeast Asian	Proportion	3,860	0.00	0.02	0.03	0.00	0.00	0.02	0.27
West Asian	Proportion	3,860	0.00	0.02	0.04	0.00	0.00	0.02	0.53
Korean	Proportion	3,860	0.00	0.02	0.03	0.00	0.00	0.02	0.28
Japanese	Proportion	3,860	0.00	0.01	0.02	0.00	0.00	0.02	0.18
Visible minorities, Not included elsewhere	Proportion	3,860	0.00	0.00	0.01	0.00	0.00	0.00	0.14
Multiple visible minorities	Proportion	3,860	0.00	0.01	0.02	0.00	0.00	0.02	0.14
Occupation categories^†^									
Frontline Occupations									
Sales & services	Proportion	3,860	0.24	0.24	0.08	0.00	0.19	0.29	0.61
Trade, transport & equip	Proportion	3,860	0.12	0.13	0.08	0.00	0.07	0.19	0.50
Nat resource, agri & prod	Proportion	3,860	0.00	0.02	0.03	0.00	0.00	0.03	0.33
Manufacturing & utilities	Proportion	3,860	0.03	0.03	0.04	0.00	0.00	0.05	0.67
Healthcare	Proportion	3,860	0.06	0.06	0.04	0.00	0.04	0.08	0.50
Other occupations									
Management	Proportion	3,860	0.11	0.11	0.06	0.00	0.07	0.15	0.50
Business, finance & admin	Proportion	3,860	0.16	0.16	0.05	0.00	0.12	0.19	0.38
Natural & applied sci	Proportion	3,860	0.06	0.07	0.04	0.00	0.04	0.09	0.33
Education, law & govt	Proportion	3,860	0.11	0.11	0.06	0.00	0.07	0.14	0.48
Art, culture & sports	Proportion	3,860	0.04	0.04	0.04	0.00	0.00	0.06	0.30

*Metrics were pooled from all DAs and summarized. ^‡^N = Number of Dissemination Area with non-missing information; Pctl. 25 = 25th percentile, Pctl. 75 = 75th percentile. ˟Visible minority subcategories are used as Other Visible Minorities in Table 1. ^†^Occupation categories are based on the National Occupation Classification (NOC). See Supplementary Table S1 for further information on census variables.

**Figure 3:**
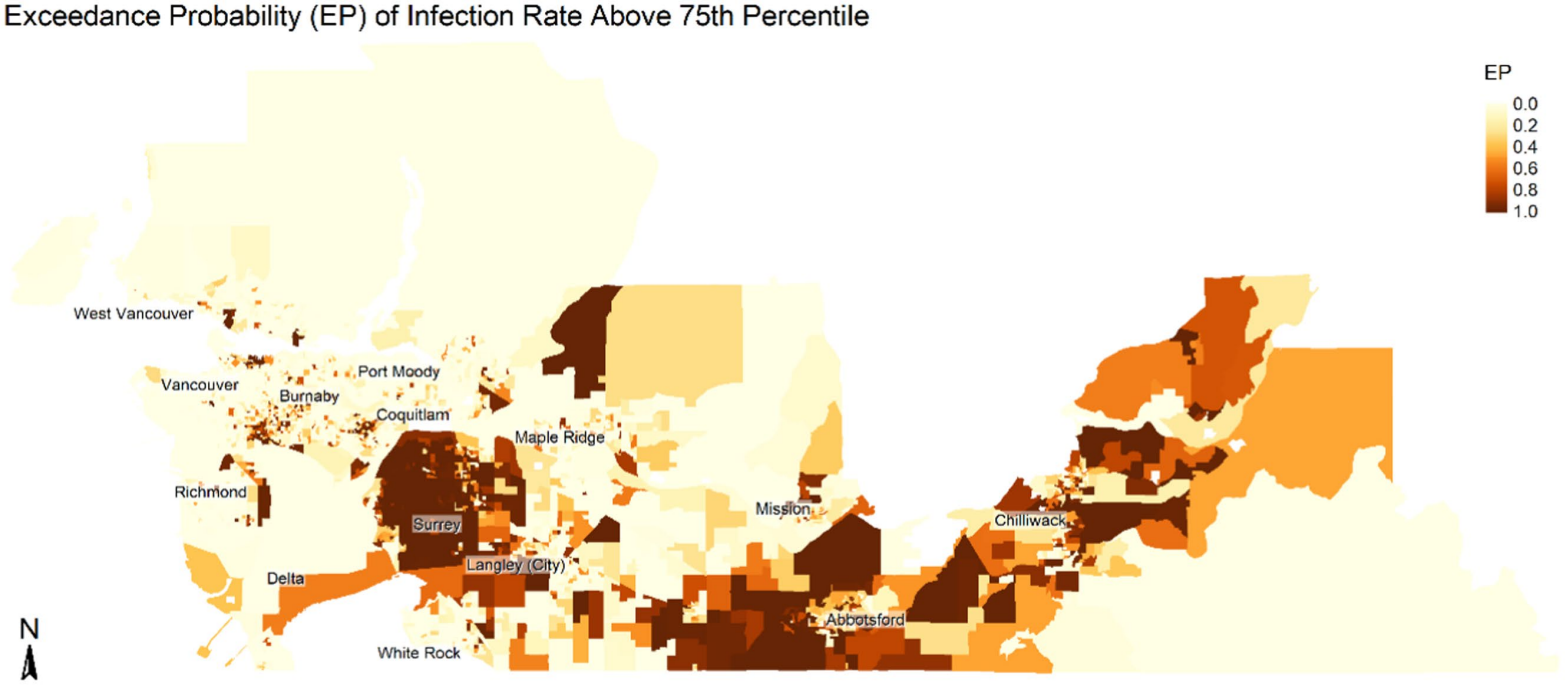
Spatial distribution of exceedance probability (EP) of infection risk being greater than 75th percentile from the BYM model. The maps show area with higher risk of COVID infection. Map is not to scale.

The results of the univariate models, model unadjusted for testing rates, and final models are shown in Table 3. The final model was adjusted for DA unemployment rate, proportion male, most common mode of commuting, proportion of recent immigrants, housing suitability, and COVID-19 testing rate. In the final model, we found an inverse relationship between the proportion of the Chinese population and risk of COVID-19 infection (RR = 0.98 95% CrI = 0.96, 0.99), whereas a higher risk was observed for the proportion of South Asian population (RR = 1.10, 95% CrI = 1.08, 1.12) and proportion of Other Visible Minority population (RR = 1.06, 95% CrI = 1.04, 1.08). Similarly, the proportion of frontline workers (RR = 1.05, 95% CrI = 1.04, 1.07) showed a positive association with the infection risks. The visible minority sub-model (Table 4) that used the different sub-groups of visible minorities showed higher infection risk in areas with a higher proportion of Black (RR = 1.13, 95% CrI = 1.05,1.21), Filipino (RR = 1.04, 95% CrI = 1.01,1.07), Arab (RR = 1.15, 95% CrI = 1.06,1.25), Southeast Asian (RR = 1.10, 95% CrI = 1.04,1.15), and West Asian population (RR = 1.07, 95% CrI = 1.01,1.12). Likewise, the occupation sub-model (Table 4) using the sub-categories of frontline workers shows a positive association between COVID-19 infections and areas that have a higher proportion of the population in occupations related to Trade, Transport, and Equipment (RR = 1.07, 95% CrI = 1.03, 1.11) and Natural Resource, Agriculture, and Production (RR = 1.07, 95% CrI = 1.01, 1.13). We also observed a wider range in the 95% credible interval in the sub-models. Figure 3 shows the spatial distribution of the exceedance probability of infection being greater than the 75th percentile from the BYM model highlighting areas at a higher risk of COVID infection in the study region.

**Table 3:** Main analysis: summary of univariate model, model unadjusted for testing rates, and final model.

Exposures and covariates	Univariate models	Model unadjusted for testing rates	Final model
	RR (95% CrI)	RR (95% CrI)	RR (95% CrI)
Key exposures			
Ethnicity (10% Increase)			
Chinese	0.96 (0.94,0.97)	0.96 (0.94,0.97)	0.98 (0.96,0.99)
South Asian	1.13 (1.12,1.15)	1.09 (1.07,1.11)	1.10 (1.08,1.12)
Other visible minorities	1.05 (1.03,1.07)	1.05 (1.03,1.08)	1.06 (1.04,1.08)
Not visible minority*	-	-	-
Occupation types (10% Increase)			
Frontline occupations	1.09 (1.07,1.11)	1.05 (1.03,1.07)	1.05 (1.04,1.07)
Non-Frontline/Other occupations*	-	-	-
Other covariates			
Unemployment rate (10% Increase)	1.07 (1.03,1.11)	1.02 (0.98,1.07)	1.05 (1.01,1.09)
Male proportion (10% Increase)	1.12 (1.06,1.17)	1.01 (0.96,1.06)	1.04 (0.99,1.09)
Commute mode (10% Increase)			
Public transport users	1.01 (0.99,1.03)	1.00 (0.97,1.02)	1.00 (0.97,1.02)
Other mode users*	-	-	-
Immigration (10% Increase)			
Recent immigrants (2011 to 2016)	1.09 (1.05,1.13)	0.99 (0.95,1.03)	1.00 (0.96,1.04)
Older immigrants/non-immigrants/non-permanent residents*	-	-	-
Housing suitability (10% increase)			
Suitable housing	0.91 (0.89,0.94)	1.02 (0.99,1.05)	0.99 (0.96,1.01)
Not suitable housing*	-	-	-
Average family size (1 person increase)	1.02 (1.02,1.02)	1.01 (1,1.01)	1.01 (1.01,1.02)
Testing rates (10% Increase)	NA	NA	1.09 (1.08,1.10)

RR, Relative Risk; 95% CrI, 95% Credible Interval. *Includes other categories in the census that contribute to the total proportion.

**Table 4:** Supplementary analysis: summary of models using subcategories of the different visible minorities and occupation types.

Models	Exposures and covariates	RR (95% CrI)
Model using subcategories of other visible minorities (Supplementary Model 1)	Chinese	0.98 (0.97,1.00)
	South Asian	1.10 (1.08, 1.12)
	Black	1.13 (1.05,1.21)
	Filipino	1.04 (1.01,1.07)
	Latin American	1.04 (0.97,1.12)
	Arab	1.15 (1.06,1.25)
	Southeast Asian	1.10 (1.04,1.15)
	West Asian	1.07 (1.01,1.12)
	Korean	0.97 (0.92,1.03)
	Japanese	0.98 (0.90,1.06)
	Visible minorities, Not included elsewhere	1.08 (0.95,1.22)
	Multiple visible minorities	1.01 (0.94,1.09)
Model using subcategories of the different occupation types (Supplementary Model 2)	Management	1.02 (0.98,1.06)
	Business, finance & admin	0.98 (0.94,1.01)
	Natural & applied science	0.97 (0.93,1.02)
	Healthcare	0.97 (0.93,1.02)
	Education, law & government	0.96 (0.93,1.00)
	Art, culture & sports	1 0.00 (0.95,1.05)
	Sales & Services	1.03 (1.00,1.07)
	Trade, transport, and equipment	1.07 (1.03,1.11)
	Natural resource, agriculture, and production	1.07 (1.01,1.13)
	Manufacturing and utilities	1.02 (0.97,1.08)

RR, Relative Risk; 95% CrI, 95% Credible Interval. Supplementary Model 1 adjusted for family size, unemployment rate, suitable housing, proportion of male population, recent immigrant, transit using population, frontline workers, and testing rates. Supplementary Model 2 adjusted for family size, unemployment rate, suitable housing, proportion of Chinese, South Asian, Other visible minorities, male population, recent immigrant, and transit using population and testing rates.

## 4 Discussion

Using ecological data, we examined the relationship between proportion of visible minorities, occupation types, and COVID-19 cases. We hypothesized that higher proportion of visible minority population and population in frontline occupations would be associated with an increased risk of COVID-19 infection, controlling for area-level sociodemographic indicators. The results of our analyses suggest COVID-19 infection risks are greater for regions with larger South Asian and Other Visible Minority populations and larger proportions of the workforce engaged in frontline work. These findings align with the existing literature on the disproportionate association between COVID-19 infection and the socio-demographic profile of the population (3, 7–10). In our study, the only factor associated with significantly lower rates of COVID-19 incidence was the larger regional Chinese population size highlighting heterogeneity in infection rates among some population groups.

Given the importance of visible minority populations and factors related to employment in our adjusted model, we further explored these effects using more detailed census population classifications. We found that a larger proportion of West Asian, Southeast Asian, South Asian, Filipino, Black, and Arab populations were associated with significantly higher rates. Conversely, only larger population proportions of Chinese were associated with significantly lower incidence. This inverse association observed in areas with higher Chinese ethnicity populations could be related to differences in rate of exposure and differential acquisition risk behaviors compared with other groups (e.g., masking and social distancing) (33). This, however, needs further investigation to understand the underlying factors for this inverse association. As expected, these estimates were associated with large uncertainty for several population groupings, given the small proportion of certain minority groups.

Replacing the derived variable ‘Frontline Workers’ with the full set of 10 occupation classifications from the census, we found that the groupings ‘Trade, Transport, and Equipment Operators’, ‘Sales and Services’, and ‘Natural Resources, Agriculture, and Production’ were associated with significantly higher risk of COVID-19 incidence. This was expected since these occupations continued to operate as usual despite a shift in remote working during the pandemic. Similar associations are observed by other studies showing higher infection rates among occupations not amenable to remote working, such as trades, transport, sales, and natural resources (9, 10). In our analysis, no occupation groups were associated with significantly lower incidence. However, the point estimates were below 1.0 for those tending to be in office and administrative settings (i.e., ‘Natural and Applied Sciences’, ‘Healthcare,’ ‘Education, Law, and Government’, and ‘Business, Finance, and Administration’). Most of these occupations favor working remotely, which minimizes exposure risks.

The spatial distribution of infection risk based on the exceedance probability of infection being greater than the 75th percentile of all DA regions (Figure 3) showed areas such as Surrey, Langley, Abbotsford, and Chilliwack having a higher concentration of neighborhoods with high infection rates. These regions also have a higher concentration of visible minority populations, such as populations of South Asian, Southeast Asian, and Filipino origins. Similarly, these regions also have a higher proportion of the population in ‘Trade, Transport, and Equipment Operators’, ‘Sales and Services’ and ‘Natural Resources, Agriculture, and Production’. Additionally, there were pockets of areas with an elevated risk of COVID-19 infection in Vancouver, Richmond, and Burnaby areas that have clusters of the visible minority population and populations engaged in frontline occupations. In addition, areas such as the Vancouver Downtown Eastside are impacted by unstable housing and socioeconomic marginalization, which impacts their incidence of COVID cases. Since a higher proportion of the visible minority populations tend to be in frontline jobs, there is an increased risk of COVID-19 infection among these populations. Furthermore, frontline jobs other than the healthcare sector may have limited occupation safety measures, increasing the risk of exposure to COVID-19 infection (34).

Our study has several limitations. The first limitation is that we only considered a single time period in our study, which limits us from making any causal inference. Therefore, our findings should be interpreted with this inherent limitation. The second limitation is the unit of analysis and making inferences at the individual level. We used the smallest standard census unit used by Census Canada to report population for the entire nation. Our study covers a comparatively large area with a sample size of 3,860 DAs, and therefore, we expect a minimal loss of information due to aggregation (35). The third limitation of our study is related to unmeasured confounding from other behavior factors and environmental factors not included in the study. Apart from these general limitations, there are specific limitations in our study related to occupation types. The ‘healthcare’ worker category does not enable differentiation with office settings such as administrative and corporate positions. Additionally, the occupation types may not truly reflect the true proportion working in each occupation since the information collected by Census Canada can also include the population not working during the week of the census count. Lastly, the findings of our study may be unique to our study areas and may not be generalizable to other study regions. Future retrospective analyses of the pre-vaccination era can address these limitations to better understand the relationship between socioeconomic factors and the spatial pattern of COVID-19 infection.

## 5 Conclusion

Our study finds that despite adjustments for variation in testing, housing, occupation, and other SES variables, there is still a substantial association between the proportion of visible minority population and frontline occupations with the risk of acquiring COVID-19 infection in British Columbia. This adds to the growing body of literature highlighting the existing disparities in the burden of diseases among different visible minority statuses. Our results highlight the role of neighborhood socio-demographic factors on COVID-19 infection. Although we cannot make individual-level inferences, the results provide evidence supporting policies targeted toward developing healthcare policies to mitigate COVID-19 infection risk among certain ethnic or vulnerable populations.

## Data availability statement

The original contributions presented in the study are included in the article/Supplementary material, further inquiries can be directed to the corresponding author.

## Ethics statement

Ethical approval was not required for the study involving humans in accordance with the local legislation and institutional requirements. Written informed consent to participate in this study was not required from the participants or the participants’ legal guardians/next of kin in accordance with the national legislation and the institutional requirements.

## Author contributions

BA: Conceptualization, Data curation, Formal analysis, Methodology, Visualization, Writing – original draft, Writing – review & editing. YA: Data curation, Writing – review & editing. NR: Writing – review & editing. FC: Writing – review & editing. SM: Data curation, Writing – review & editing. CR: Writing – review & editing. NJ: Conceptualization, Writing – review & editing. MO: Conceptualization, Formal analysis, Supervision, Writing – review & editing. MI: Formal analysis, Methodology, Software, Supervision, Writing – original draft, Writing – review & editing.

## Footnotes

^1^https://bccdc.shinyapps.io/covid_geospatial_model_shiny_app/
