# Supplementary material for: Visible minority status and occupation were associated with increased COVID-19 infection in Greater Vancouver British Columbia between June and November 2020: an ecological study
Source: Front Public Health. 2024 Feb 28;12:1336038. doi: 10.3389/fpubh.2024.1336038 (PMC10935735; doi:10.3389/fpubh.2024.1336038)
Supplement: Supplementary file 1 [file Table_1.DOCX]

Supplementary Material

Table S1. Census variables used in the analysis

| Variables Used In The Models | Variables In Census (Variable Code Followed By Description) | Reference |
| --- | --- | --- |
| Visible Minorities | V1405 Total - Visible Minority For The Population In Private Households - 25% Sample Data* |  |
|  | V1406 Total Visible Minority Population | [https://www12.statcan.gc.ca/census-recensement/2016/ref/dict/pop127-eng.cfm https://www23.statcan.gc.ca/imdb/p3VD.pl?Function=getVD&TVD=257518&CVD=257519&CPV=1&CST=19102015&CLV=1&MLV=2](https://www23.statcan.gc.ca/imdb/p3VD.pl?Function=getVD&TVD=257518&CVD=257519&CPV=1&CST=19102015&CLV=1&MLV=2) |
|  | V1407 South Asian |  |
|  | V1408 Chinese |  |
|  | V1409 Black |  |
|  | V1410 Filipino |  |
|  | V1411 Latin American |  |
|  | V1412 Arab |  |
|  | V1413 Southeast Asian |  |
|  | V1414 West Asian |  |
|  | V1415 Korean |  |
|  | V1416 Japanese |  |
|  | V1417 Visible Minority, N.I.E. |  |
|  | V1418 Multiple Visible Minorities |  |
| Occupation Types | V1984 Total Labour Force Population Aged 15 Years And Over By Occupation - National Occupational Classification (Noc) 2016 - 25% Sample Data* | <https://www12.statcan.gc.ca/census-recensement/2016/ref/dict/pop157-eng.cfm> |
|  | V1985 Occupation - Not Applicable |  |
|  | V1986 All Occupations |  |
|  | V1987 0 Management Occupations |  |
|  | V1988 1 Business, Finance And Administration Occupations |  |
|  | V1989 2 Natural And Applied Sciences And Related Occupations |  |
|  | V1990 3 Health Occupations |  |
|  | V1991 4 Occupations In Education, Law And Social, Community And Government Services |  |
|  | V1992 5 Occupations In Art, Culture, Recreation And Sport |  |
|  | V1993 6 Sales And Service Occupations |  |
|  | V1994 7 Trades, Transport And Equipment Operators And Related Occupations |  |
|  | V1995 8 Natural Resources, Agriculture And Related Production Occupations |  |
|  | V1996 9 Occupations In Manufacturing And Utilities |  |
| Suitable Housing | V1722 Total - Private Households By Housing Suitability - 25% Sample Data* | <https://www12.statcan.gc.ca/census-recensement/2016/ref/dict/households-menage029-eng.cfm> |
|  | V1723 Suitable |  |
|  | V1724 Not Suitable |  |
| Average Family Size | V0155 Average Size Of Census Families* | <https://www12.statcan.gc.ca/census-recensement/2016/ref/dict/fam036-eng.cfm> |
| Recent Immigration | V1222 Total - Immigrant Status And Period Of Immigration For The Population In Private Households - 25% Sample Data* | <https://www12.statcan.gc.ca/census-recensement/2016/ref/dict/pop133-eng.cfm> |
|  | V1231 2011 To 2016 |  |
| Public Transport | V2030 Total - Main Mode Of Commuting For The Employed Labour Force Aged 15 Years And Over In Private Households With A Usual Place Of Work Or No Fixed Workplace Address - 25% Sample Data* | <https://www12.statcan.gc.ca/census-recensement/2016/ref/dict/pop177-eng.cfm> |
|  | V2033 Public Transit |  |
| Unemployment | V1972 Unemployment Rate | <https://www12.statcan.gc.ca/census-recensement/2016/ref/dict/pop125-eng.cfm> |
| Male Proportion | V0006 Total - Age Groups And Average Age Of The Population - 100% Data* | <https://www23.statcan.gc.ca/imdb/p3VD.pl?Function=getVD&TVD=62207> |
|  | V0032 Male - Age Groups And Average Age Of The Population - 100% Data |  |
| *Note: * Represents denominator used To calculate proportion* | | |
